# Thermal Tolerance in the Cellophane Bee *Colletes inaequalis* Reflects Early Spring Adaptation and Is Independent of Body Size and Sex

**DOI:** 10.1002/ece3.71983

**Published:** 2025-08-12

**Authors:** Victor H. Gonzalez, Natalie Herbison, Andres Herrera, Kennan Oyen, Deborah R. Smith

**Affiliations:** ^1^ Department of Ecology and Evolutionary Biology University of Kansas Lawrence Kansas USA; ^2^ Animal Diseases Research Unit Agricultural Research Service, United States Department of Agriculture Pullman Washington USA

**Keywords:** chill coma, CT_Max_, CT_Min_, ground‐nesting, honey bees, pollinators, protandry

## Abstract

*Colletes inaequalis* Say is a univoltine, ground‐nesting solitary bee and one of the first pollinators to emerge in the North American springtime. Males emerge earlier and are smaller than females. Despite its role as a pollinator of early spring wild plants and crops, the thermal ecology of *C. inaequalis* remains unexplored. We assessed its lower (CT_Min_) and upper (CT_Max_) critical thermal limits and chill coma recovery time, testing the effects of sex and body size (fresh body mass and intertegular distance, ITD). We also compared these thermal traits to those of the European honey bee ( *Apis mellifera* ), a similarly sized species active during the same season. Given that *C. inaequalis* males sometimes emerge before floral resources are available and that snowfall can occur after their emergence, we examined whether food deprivation and repeated or prolonged cold exposure impair their ability to recover from chill coma. We found that males and females of *C. inaequalis* exhibit similar thermal limits, and neither CT_Min_ nor CT_Max_ is influenced by ITD. Body mass did not affect chill coma recovery time. *Colletes inaequalis* was significantly more cold‐tolerant but less heat‐tolerant than honey bees, recovering much faster from chill coma. Repeated cold exposure significantly impacted recovery time, while food availability was the primary factor influencing male survival. These findings suggest that *C. inaequalis* is physiologically adapted to early spring conditions, in contrast to honey bees, which likely rely on social and behavioral mechanisms to cope with low temperatures. Our results suggest that bee communities may exhibit thermal tolerances that align with their seasonal activity periods.

## Graphical abstract

The cellophane bee, *Colletes inaequalis* , is a ground‐nesting, solitary species and key pollinator of spring plants. Our study shows it is thermally adapted to early spring conditions, with similar thermal traits in males and females despite differences in body size and emergence timing. Repeated cold exposure impairs recovery, and starvation increases mortality risk.

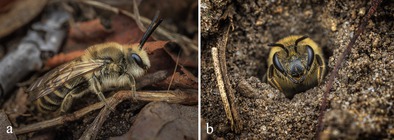

## 1 Introduction

The importance of bees as the main pollinators of wild and cultivated plants, as well as the increasing threats they face due to anthropogenic stressors, is undeniable (Wagner 2020; Cornelisse et al. 2025). Bees are exposed to numerous stressors that include habitat loss, pathogens, and pesticides, with climate change as the leading factor (Goulson et al. 2015; López‐Uribe 2021). Thus, an interest in understanding the thermal biology of bees is rapidly increasing, as such knowledge might prove useful in predicting their potential responses to climate change.

There are over 20,000 bee species worldwide, and most are solitary (Michener 2007; Danforth et al. 2019). Despite their ecological and economic importance, the thermal biology of bees remains largely understudied, with existing research showing strong geographic and phylogenetic biases (Gonzalez, Oyen, Ávila, et al. 2022; Dillon et al. 2025). Most studies have been conducted in North America and Europe and have disproportionately focused on highly social species, such as bumble bees (Bombini, genus *Bombus*) (Oyen and Dillon 2018; Gonzalez, Oyen, Aguilar, et al. 2022), honey bees (Apini, genus *Apis*) (Gonzalez, Oyen, Ávila, et al. 2022), and several genera of stingless honey‐making bees (Meliponini) (Gonzalez, Oyen, Vitale, et al. 2022; Nacko et al. 2023). While some data exist for thermal traits of solitary species (e.g., Bishop and Armbruster 1999; Gonzalez et al. 2020; Barrett et al. 2023; Gudowska and Morón 2024; Herrera et al. 2023; Jones et al. 2024; DeHaan et al. 2025; Ratoni et al. 2025), the emphasis on social taxa is likely due to their large colony sizes, their extended or even perennial colony cycles, and their accessibility, as some are already managed and commercially available (Michener 2007). However, these highly social bees represent a small fraction of global bee diversity and are phylogenetically clustered within the corbiculate Apidae. Furthermore, they can thermoregulate their nests (Roubik 2006), unlike solitary bees, which must instead rely on other behavioral mechanisms and physiological adaptations to cope with environmental fluctuations (Ostwald et al. 2024). Consequently, the patterns documented to date in bees' thermal biology may not accurately reflect broader patterns for bees in general, given the shared phylogenetic and life history traits of social species (Dillon et al. 2025).

Insect thermal tolerance is influenced by a myriad of biotic and abiotic factors, including elevation, body size, age, sex, and nutrition (e.g., Chidawanyika et al. 2017; Sunday et al. 2019; González‐Tokman et al. 2021), some of which have been explored in social bees. For example, heat tolerance tends to increase with body size across species, including certain species of bumble bees and stingless bees, but shows mixed responses to elevation at the population and community level, with some species exhibiting no change or even lower heat tolerance at higher altitudes when compared to low‐elevation populations or communities (Oyen et al. 2016; Gonzalez, Oyen, Aguilar, et al. 2022; Gonzalez, Oyen, Vitale, et al. 2022; Gonzalez et al. 2024). However, studies on solitary bees or species with alternative social structures remain scarce, with only a handful of species examined to date (e.g., Gonzalez et al. 2020, 2023, 2024; Da Silva et al. 2021; Barrett et al. 2023; Jones et al. 2024). This knowledge gap hinders our understanding of thermal adaptation in most bee species and limits our ability to predict their responses to climate change.

**FIGURE 1:**
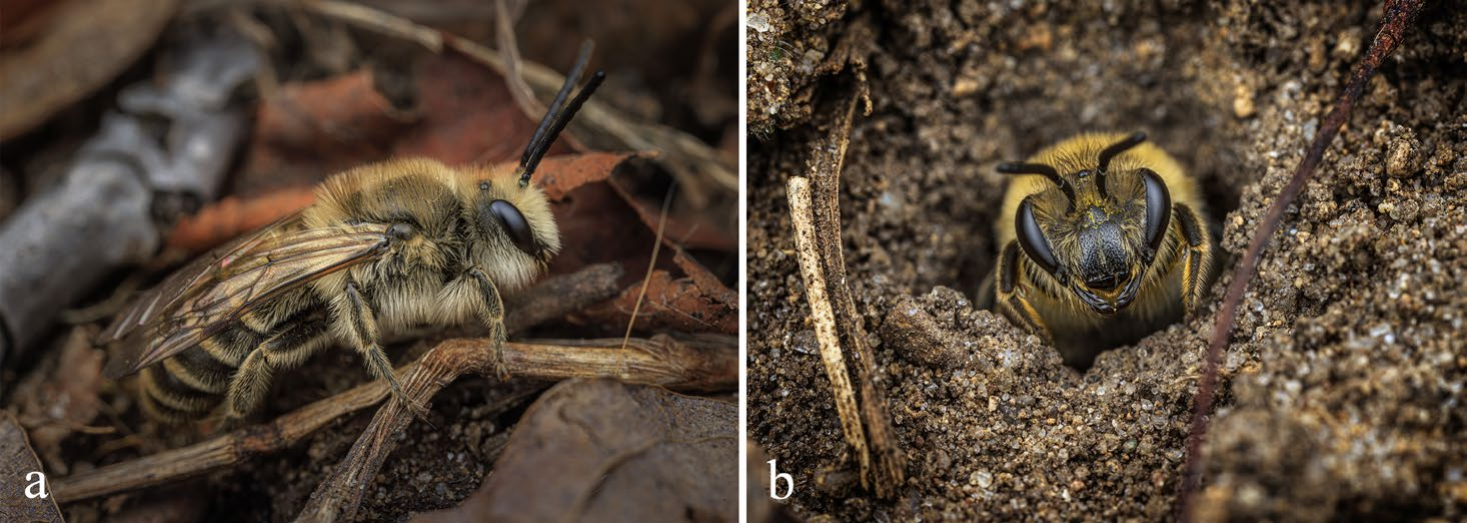
The cellophane bee *Colletes inaequalis* Say at a nesting aggregation in Tenhave Woods Nature Preserve in Royal Oak, Michigan, USA. (a) Newly emerged male on March 13, 2024. (b) Female at the nest entrance on April 6, 2024. Photographs by Joseph Ferraro (https://www.joseph‐ferraro.com/).

Here, we investigate the thermal ecology of *Colletes inaequalis* Say (Colletidae), a common ground‐nesting solitary bee and one of the first species to emerge in the North American spring (Figure 1). Despite its importance as a wild pollinator of early spring plants and its potential role in crop pollination (Batra 1980, 1995; Gardner and Ascher 2006), key aspects of its thermal ecology remain unexplored. Understanding the thermal biology of spring pollinators, such as *C. inaequalis* , is essential for predicting the potential effects of climate change on their survival and ecological interactions (e.g., Kudo and Ida 2013; Gérard et al. 2020). Like most solitary bees, *C. inaequalis* is univoltine, producing a single generation per year. Males emerge approximately 2 weeks before females, often flying when snow is still on the ground and floral resources are scarce or absent (Batra 1980). Additionally, males are significantly smaller than females (see Section 3). These differences in phenology and body size suggest that males and females may differ in their thermal tolerance, warranting further study.

We assessed the thermal tolerance of *C. inaequalis* using its critical thermal limits, the minimum (CT_Min_) and maximum (CT_Max_) temperatures at which an animal can maintain muscle control (Lutterschmidt and Hutchison 1997). We also evaluated chill coma recovery time, the time required for an individual to regain mobility after experiencing a chill coma (Sinclair et al. 2015; Roeder and Daniels 2022). While critical thermal limits indicate the thermal extremes an organism can withstand, chill coma recovery time provides insight into an individual's ability to recover from acute cold stress, an increasingly relevant factor as extreme cold events become more frequent, prolonged, and intense due to climate change (IPCC 2021; Harvey et al. 2023). Furthermore, given anecdotal observations that males sometimes emerge before floral resources are available or that snowfall can occur after their emergence (e.g., Batra 1980; V.H.G., personal observations), we also examined whether food deprivation and repeated or prolonged cold exposure impair the ability of *C. inaequalis* males to recover from chill coma.

Given that males emerge earlier and are likely exposed to lower ambient temperatures than the females, we hypothesize that males will exhibit greater cold tolerance while maintaining comparable heat tolerance. This expectation would be consistent with the thermal adaptation hypothesis, which predicts that the thermal limits of an organism are shaped by the thermal conditions of its local environment (Sunday et al. 2012). Because smaller bees have a higher surface‐area‐to‐volume ratio, they gain and lose heat more quickly than larger bees through increased convective heat transfer (Heinrich and Heinrich 1983; Oyen et al. 2016). Thus, we also predict that CT_Min_ will decrease and CT_Max_ will increase with increasing body size, respectively, indicating a greater range of thermal tolerance in larger individuals. In addition, we predict that longer exposure to cold stress, repeated cold exposure, and starvation will extend chill coma recovery time, reflecting the physiological costs of these stressors. Finally, we compared these thermal traits to those of the European honey bee ( *Apis mellifera* L.), a similarly sized species that frequently forages during early spring at our study site. Given its earlier emergence and active foraging period restricted to the spring season, we hypothesize that *C. inaequalis* will exhibit greater cold tolerance but lower heat tolerance than honey bees.

## 2 Material and Methods

### 2.1 Species Synopsis

*Colletes inaequalis* is a polylectic, solitary, gregarious bee with a univoltine life cycle, found across the eastern United States and Canada. These bees nest underground in bare soil, typically at depths of 7–39 cm but with records of up to 86 cm, with each nest containing up to seven cells (Batra 1980). This species exhibits strong site fidelity or philopatry, with offspring often nesting within the same aggregation as their parents. Males emerge in early spring (Figure 1a) and patrol nesting sites for mates for about 2 weeks before females emerge, after which copulation occurs, and females begin digging nests and provisioning brood cells (Figure 1b). Adult activity typically ceases by early summer, as larvae develop into prepupae. Although *C. inaequalis* is likely to overwinter as adults, the exact overwintering stage remains uncertain (Batra 1980). As an early spring bee, *C. inaequalis* plays a key role in the pollination of economically important crops such as apples and blueberries (Batra 1995; Gardner and Ascher 2006).

### 2.2 Study Site and Bee Collections

We used both males and females of *C. inaequalis* captured between March and April 2022–2024 from seven nesting aggregations located at the campus of the University of Kansas, Lawrence, Kansas, USA (38.959796°N, −95.246428°W, 290–316 m). Nesting aggregations were separated from each other by 300–450 m and have been occupied by this species at least since 2016 (D.R.S., personal observations). We collected bees with a net when activity was highest, between 10:00 and 12:00 h, and transferred them individually to plastic containers, which we capped with fabric (1 mm mesh). We collected honey bee foragers for comparison from flowers around the nesting aggregations, as well as from artificial feeders placed near two Langstroth hives kept within the city limits by one of us (D.R.S.) about 2 km south of the university campus. We transported bees to the laboratory in a cooler, and all bioassays were conducted within 1–2 h of collection. Each assay described below was performed on a separate set of bees. Given that collections were opportunistic, sample sizes varied across species, sexes, and treatments (see summary Tables 1 and 2).

**TABLE 1:** Intertegular distance (ITD), fresh body mass (FBM), critical thermal minima (CT_Min_), and maxima (CT_Max_), and chill coma recovery time (CCRT) after 2‐h exposure to 0°C, measured for *Colletes inaequalis* and *Apis mellifera* . The mean value is followed by standard error (±) and sample size.

Trait	*Colletes inaequalis*		*Apis mellifera*
	Male	Female	Worker
ITD (mm)	2.42 ± 0.02, *n* = 73	3.04 ± 0.02, *n* = 59	3.00 ± 0.02, *n* = 40
FBM (mg)	56.83 ± 2.75, *n* = 18	90.78 ± 2.94, *n* = 23	103.18 ± 3.35, *n* = 34
CT_Min_ (°C)	4.69 ± 0.19, *n* = 71	5.42 ± 0.16, *n* = 58	10.54 ± 0.27, *n* = 40
CT_Max_ (°C)	44.36 ± 0.20, *n* = 65	43.10 ± 0.29, *n* = 57	45.95 ± 0.32, *n* = 31
CCRT (min)	7.40 ± 0.36, *n* = 18	6.74 ± 0.18, *n* = 23	12.08 ± 0.73, *n* = 34

**TABLE 2:** Effects of cold prolonged and repeated exposure and starvation on chill coma recovery (min) in *Colletes inaequalis* males. To establish a baseline for chill coma recovery time, all individuals were exposed to 0°C for either 1 h (initially 31 males) or 2 h (initially 33 males) upon collection. After this initial assessment (timepoint 0), approximately half of the males were maintained under fed conditions, while the other half were deprived of food for the duration of the experiment. Cold exposure (1 or 2 h) was repeated at 24‐h intervals, matching the initial exposure duration, for a total of four timepoints: 0, 24, 48, and 72 h. Chill coma recovery time (mean ± SE and sample size) was recorded following each cold exposure. Decreasing sample sizes across timepoints reflect mortality over the course of the experiment.

Repeated cold exposure (h)	Cold exposure duration/Feeding condition			
	1 h		2 h	
	Fed	Unfed	Fed	Unfed
0	7.58 ± 0.82, *n* = 15	6.00 ± 0.40, *n* = 16	7.28 ± 0.77, *n* = 16	6.70 ± 0.50, *n* = 17
24	9.07 ± 0.86, *n* = 11	8.91 ± 1.13, *n* = 14	9.00 ± 0.98, *n* = 14	8.76 ± 0.58, *n* = 15
48	7.67 ± 1.07, *n* = 10	9.00 ± 1.41, *n* = 6	11.86 ± 1.43, *n* = 14	12.91 ± 1.85, *n* = 7
72	9.03 ± 0.80, *n* = 8	—	10.42 ± 1.07, *n* = 13	11.43 ± 3.70, *n* = 3

To characterize the ambient temperature and humidity experienced by *C. inaequalis* at the nesting aggregation, we placed iButton data loggers (weight: 3.104 g; DS1923 Hygrochron; Maxim Integrated, San Jose, California) at two arbitrarily selected nesting sites. As in Gonzalez et al. (2020), we protected each datalogger from solar radiation with aluminum foil and hung them 1 m above ground from shrubs we found next to the nesting site. We recorded temperature and humidity every 30 min for 41 consecutive days, starting on March 30 and ending on May 9, 2023.

### 2.3 Critical Thermal Limits

We measured bees' critical thermal limits using a dynamic (ramping temperature) protocol with the Elara 2.0 (IoTherm, Laramie, WY), a fully programmable heating/cooling anodized aluminum stage designed for precision temperature control of laboratory and field samples. We modified the stage with a Styrofoam cooler and clear acrylic lid to minimize the impact of airflow across the aluminum sample stage and maintain temperature stability across all vials. We placed bees individually inside glass vials (9 × 30 mm, 0.92 cm^3^) plugged with a cotton ball. As in Gonzalez, Oyen, Ávila, et al. (2022), we used an initial temperature of 22°C and held bees for 10 min at this temperature before increasing it or decreasing it at a rate of 0.5°C min^−1^. The rate of temperature change during thermal assays is known to influence estimates of critical thermal limits, and it varies significantly among studies (e.g., Terblanche et al. 2007; Gonzalez, Oyen, Ávila, et al. 2022). We used a recommended medium rate of temperature change (0.5°C min^−1^) to reduce the time required for each assay, minimize the influence of confounding physiological stressors, and facilitate comparison across species (Gonzalez, Oyen, Ávila, et al. 2022). We placed vials horizontally on the stage to avoid bees climbing up a vertical vial wall, away from the source of heating or cooling. To estimate the temperature inside the vials, we placed a K‐type thermocouple inside two empty glass vials plugged with a cotton ball. We individually tracked these vial temperatures using a TC‐08 thermocouple data logger (Pico Technology, Tyler, TX, USA). We acknowledge that the absence of a bee in these vials may result in slight deviations from the actual thermal conditions experienced during assays, as bees can produce or retain heat, thus influencing internal vial temperatures during heating or cooling. Oyen and Dillon (2018) demonstrated that while bumble bee body temperatures track vial temperatures closely, the difference between the two becomes more pronounced when bees were cooled or heated. Given this, and the potential for variation across species and body sizes, we report vial temperatures as an estimate of the thermal conditions experienced by bees, an approach consistent with other thermal studies. As an approximation of bees' thermal limits, we used the temperature at which bees show signs of curling up (CT_Min_, Oyen and Dillon 2018) or loss of muscular control, spontaneously flipping over onto their dorsa and spasming (CT_Max_, Lutterschmidt and Hutchison 1997). These behavioral indicators are stereotypical across bee species and yield results comparable to those obtained using more precise methods and equipment, such as respirometry (Oyen and Dillon 2018; Barrett et al. 2023). Once the assays concluded, we euthanized all individuals to measure their intertegular distance (ITD) as indicated below. Voucher specimens are in the Snow Entomological Museum, University of Kansas, Lawrence, KS.

### 2.4 Chill Coma Recovery Time

We assessed chill coma recovery in bees after a 2‐h exposure to 0°C. This temperature and duration were chosen because they fall below the CT_Min_ of *C. inaequalis* while remaining short enough to avoid significant negative effects on honey bees. Pilot assays indicated that longer exposures at this temperature increased honey bee mortality. We placed each bee individually in a plastic vial plugged with a cotton ball and inserted it into a thick layer of granulated ice stored in a cooler, ensuring the vial's entrance remained uncovered. We used an empty plastic vial with a thermocouple to monitor the temperature inside the vial during the duration of the experiment. After the exposure period, we removed the bees from the ice, placed them on a paper towel at room temperature (22°C), and recorded their recovery using a Sony Handycam video recorder mounted on a tripod. We analyzed videos using a personal computer to determine the time each bee took to right itself, serving as a proxy of chill coma recovery. To minimize any impact on the local population of *C. inaequalis* , we measured the fresh body mass of bees upon capture (see below) and released them after the experiment.

We chose to assess cold tolerance in *C. inaequalis* using CT_Min_ and chill coma recovery time, as these thermal traits reflect sublethal responses to cold exposure and provide insight into important ecological thresholds where bees can no longer feed, reproduce, or avoid predation (David et al. 1998). CT_Min_ is a reversible state of paralysis known as chill coma and is driven by a loss of homeostasis (Hazell and Bale 2011). Recovery following chill coma depends on the restoration of homeostasis but also the duration of the exposure (Macmillan et al. 2012). In contrast, traits such as supercooling or freezing points measure the temperature at which body fluids spontaneously freeze, a much lower threshold than CT_Min_ and typically a lethal event for non‐freeze‐tolerant species (e.g., Sinclair et al. 2015).

### 2.5 Effects of Cold Exposure and Starvation on Chill Coma Recovery in Male *C. inaequalis*

To assess the effects of cold exposure duration, repeated cold exposure, and starvation on chill coma recovery, we conducted an experiment on *C. inaequalis* males in which we varied both the duration of cold exposure and feeding conditions. Upon field collection, we first established a baseline recovery time for all individuals following either 1‐ or 2‐h exposure to 0°C. After this initial assessment, bees were randomly assigned to one of two treatment groups: one provided with food and one deprived of food (hereafter “unfed”). Bees in the fed group received ad libitum access to a 50% sucrose solution via a moistened cotton ball inside the plastic vial, which we replaced daily after each coma recovery trial. All bees were then subjected to daily cold exposure at 0°C, either 1‐ or 2‐h durations that matched their initial baseline exposure and recorded chill coma recovery time over three consecutive days. Between trials, we kept bees at room temperature (20°C–22°C, 60%–70% RH) inside a closed cardboard box with a water‐filled open container to ensure humidity. We recorded mortality daily prior to each cold exposure trial. We measured bees' fresh body mass upon collection and assessed chill coma recovery time as described above. To minimize potential temporal effects on the recovery time, we assessed this thermal trait around noon each day.

### 2.6 Operative Temperatures

To place the results of the thermal assays in an ecological context, we also measured the operative temperature of *C. inaequalis* under natural field conditions. This temperature represents the temperature of an organism at equilibrium with its environment, integrating the effects of air temperature, radiative exchange, cuticular water loss, and convection, without the influence of behavioral thermoregulation, metabolic heating, or evaporative cooling (Johnson et al. 2023). As in Naumchik and Youngsteadt (2023), we used a dead, dried *C. inaequalis* male with a K‐type thermocouple inserted into its thorax. We placed the specimen at about 5 cm above the ground on top of a dandelion flower ( *Taraxacum officinale* ), near a nesting aggregation. We recorded temperature every second for five consecutive days, starting on March 15, 2024 when males were already flying. Simultaneously, we recorded ambient air temperature under the shade by placing a thermocouple inside a solar radiation shield positioned about the same height above ground. Both temperatures were individually recorded using a Hobo data logger. Operative temperatures were typically more extreme than shaded ambient air temperatures and occasionally exceeded the bees' physiological thermal limits (see Section 3), as the specimen was fully exposed to solar radiation and convective effects. Thus, these measurements more accurately represent the thermal environment experienced by bees in the field.

### 2.7 Body Size

We measured the minimum ITD, a robust predictor of bee dry mass commonly used in ecological studies (Cane 1987; Kendall et al. 2019), along with fresh body mass as proxies of body size. We measured ITD for all specimens used in the critical thermal assays using an ocular micrometer on an S6E stereomicroscope (Leica Microsystems, Wetzlar, Germany). We measured fresh body mass for all specimens used in the chill coma recovery bioassays. To determine fresh body mass, we weighed each bee inside a plastic vial using a precision balance (Ohaus Pioneer PX163/E, Pine Brook, New Jersey; ±0.001 g resolution) and subtracted the weight of the empty vial. We measured fresh body mass in the chill coma recovery time assays because we released bees after the experiment.

### 2.8 Statistical Analyses

We conducted statistical analyses in R (R Core Team 2025). To test for differences in ambient temperature and relative humidity between nesting aggregations, we used a one‐way ANOVA model with the lm function. The same function was used to implement a linear regression analysis to explore the relationship between ambient temperature and humidity. To test for differences in thermal tolerance between sexes of *C. inaequalis* , we performed an ANCOVA while controlling body size. Using each thermal trait (CT_Min_, CT_Max_, and recovery time) as a response variable, we implemented a linear mixed‐effect model (LMM) with the lmer function in the lme4 package (Bates et al. 2015). We included sex (male or female) as a fixed factor, collection date as a random factor, and body size (either ITD or body mass) as a covariate. To assess differences in thermal tolerance between *C. inaequalis* and honey bees, we implemented similar LMMs for each thermal trait, pooling data for *C. inaequalis* and using species ( *C. inaequalis* and honey bees) as a fixed factor. We evaluated the relationship between body size (either ITD or body mass) and each thermal trait using a linear regression analysis with the lm function. To assess the effect of cold exposure duration, repeated cold stress, and starvation on *C. inaequalis* males' chill coma recovery, we implemented an LMM with recovery time as the response variable. Duration (1 or 2 h), feeding condition (fed versus unfed), measuring time (hours post‐exposure: 0, 24, 48, and 72 h post‐exposure) were included as fixed factors, collection date as a random factor, and fresh body mass as a covariate. We initially used a model including interactions among factors, but because these were not significant, we simplified the model by removing them. We assessed the significance of fixed effects using a Type II Wald *χ* ^2^ test with the car package (Fox and Weisberg 2019). To assess differences in survival under different exposure durations, repeated cold exposure, and starvation, we used failure‐time analyses. We implemented a Cox proportional hazard model using the survival package (Therneau 2024), including exposure duration (1 or 2 h) and feeding condition (fed versus unfed) as fixed factors, with collection date as a random effect.

## 3 Results

### 3.1 Ambient Temperature and Relative Humidity

Temperature and relative humidity varied greatly both daily and throughout the monitoring period (Figure S1a,b), and did not differ significantly between the two nesting aggregations (ANOVA, Temperature: *χ* ^2^ = 0.032, DF = 1, *p* = 0.86; Relative humidity: *χ* ^2^ = 1.55, DF = 1, *p* = 0.21). These variables were inversely correlated (*p* < 0.001, *R* ^2^ = 0.082; Figure S1c). Mean daily temperatures ranged from a low of 8.41°C in early April to a high of 26.33°C in early May, with observed extremes reaching a minimum of 0.44°C and a maximum of 36.4°C during the monitoring period (Median: 15.06°C; Figure S1d). Similarly, the mean daily relative humidity ranged from 25.04% to 91.43%, with absolute values varying between a minimum of 9.08% and a maximum of 100% during the monitoring period (Median: 49.61%; Figure S1d).

### 3.2 Thermal Tolerance of *C. inaequalis*

**FIGURE 2:**
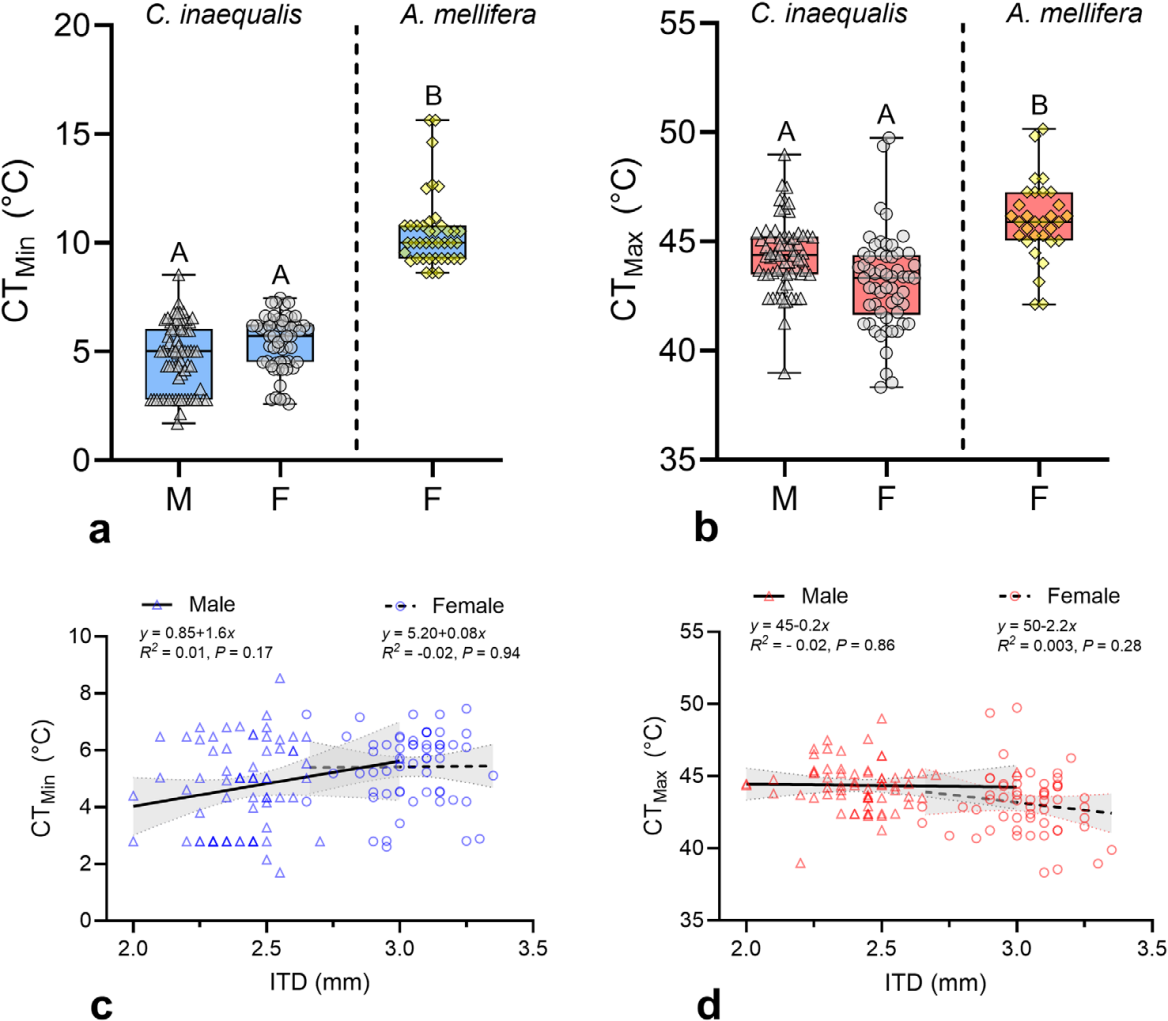
Critical thermal minima (CT_Min_) and maxima (CT_Max_) of *Colletes inaequalis* and *Apis mellifera* , and their relationship to intertegular distance (ITD) in *C. inaequalis* . (a,b) Boxplots display the median, quartiles, and extreme values. For each figure, different letters above the boxplots indicate significantly different means (*p <* 0.05). (c, d) Relationship between ITD and CT_Max_ for males and females. Trend lines represent linear regressions, and the gray areas around the lines are 95% confidence intervals.

Females were, on average, about 20% larger and 37% heavier than males (Table 1), and these differences were significant (ITD: Wald *χ* ^2^ = 11.73, FBW: *χ* ^2^ = 67.8; DF = 1, *p <* 0.001 in both cases). Neither CT_Min_ nor CT_Max_ differed between sexes after accounting for body size (ANCOVA, CT_Min_ *χ* ^2^ = 0.28, DF = 1, *p* = 0.60; CT_Max_ *χ* ^2^ = 0.71, DF = 1, *p* = 0.40; Figure 2a,b). In both sexes, CT_Min_ and CT_Max_ did not significantly change with increasing ITD (Figure 2c,d).

**FIGURE 3:**
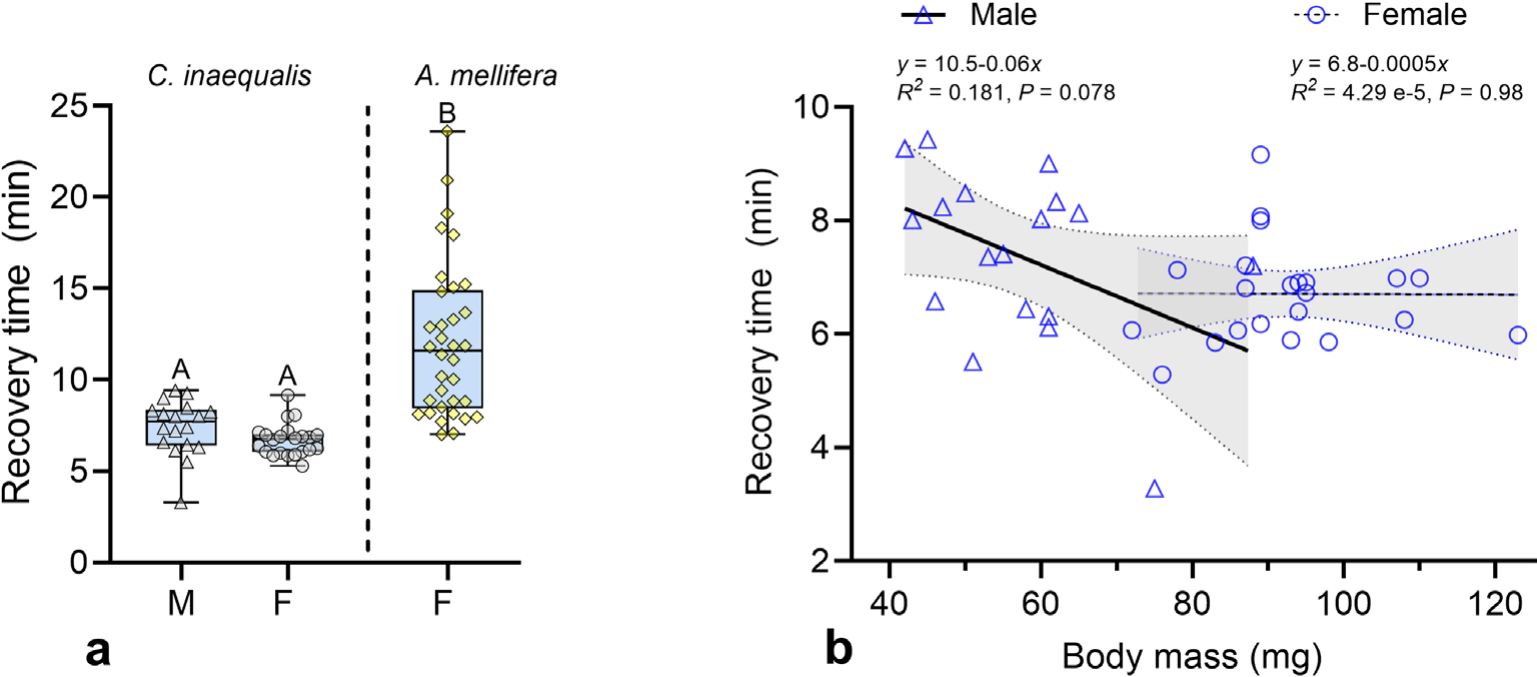
Chill coma recovery time of *Colletes inaequalis* and *Apis mellifera* following a 2‐h exposure to 0°C, and its relationship to fresh body mass in males and females of *C. inaequalis* . (a) Boxplots display the median, quartiles, and extreme values. Different letters above the boxplots indicate significantly different means (*p <* 0.05). (b) Trend lines represent linear regressions, and the gray areas around the lines are 95% confidence intervals.

Similarly, chill coma recovery time did not differ between sexes after accounting for body size (*χ* ^2^ = 0.032, DF = 1, *p* = 0.86; Figure 3a) and did not significantly change with body mass for either male or female (Figure 3b).

### 3.3 Operative Temperatures of *C. inaequalis*

Operative temperatures experienced by *C. inaequalis* ranged from −8.58°C to 44.7°C (Median: 7.01 ± 0.018, *n* = 114.5 h), and were on average 0.26°C higher than ambient temperatures (median: 7.67 ± 0.014, −5.81°C to 30.5°C, *n* = 114.5 h). While peak operative temperatures during the monitoring period often remained well below bees' CT_Max_, minimum values frequently approached or dropped below CT_Min_ (Figure S2).

### 3.4 Comparison With Honey Bees

*Colletes inaequalis* displayed a CT_Min_ 5.1°C–5.9°C lower than honey bees' foragers, and this difference was significant after accounting for body size (*χ* ^2^ = 287.5, DF = 2, *p* < 0.001; Figure 2a). Similarly, *C. inaequalis* had a lower CT_Max_, averaging 1.6°C–2.9°C below that of honey bees, with this difference also being significant after accounting for body size (*χ* ^2^ = 6.99, DF = 2, *p* = 0.030; Figure 2b). In addition, *C. inaequalis* recovered from chill coma about twice as fast as honey bees following a 2‐h exposure to 0°C (*χ* ^2^ = 15.32, DF = 1, *p* < 0.001; Figure 3a).

### 3.5 Cold Exposure and Starvation on Chill Coma Recovery in Male *C. inaequalis*

**FIGURE 4:**
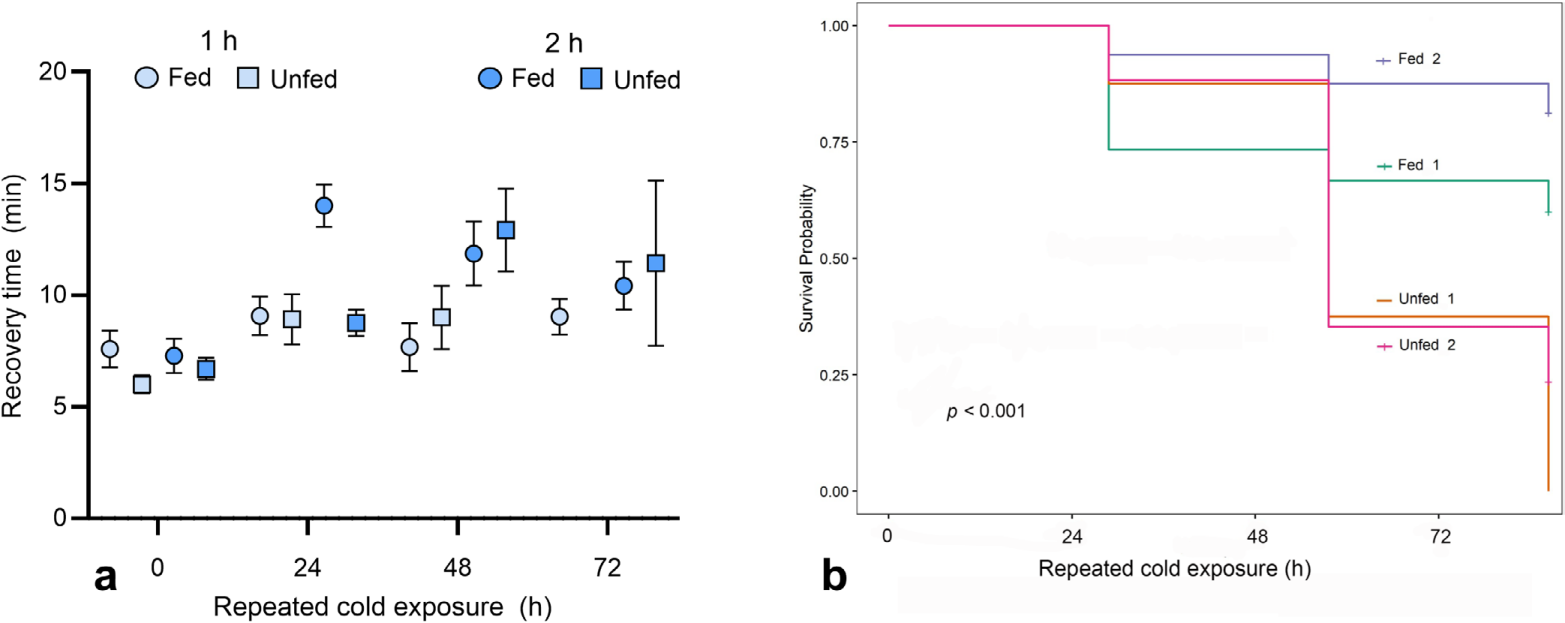
Effects of prolonged and repeated cold exposure and starvation on chill coma recovery in *Colletes inaequalis* males. (a) Chill coma recovery time (mean ± SE) following 1‐ or 2‐h cold exposure to 0°C in fed and unfed males, with recovery measured at 24‐h intervals, matching the initial exposure duration, for a total of four timepoints: 0, 24, 48, and 72 h (see details in Table 2). (b) Kaplan–Meier survival curves of fed and unfed individuals exposed to either 1‐ or 2‐h cold treatments at 0°C. Mortality was recorded daily prior to each cold exposure trial.

After controlling for body size, neither exposure duration (1 vs. 2 h) (*χ* ^2^ = 3.01, DF = 1, *p* = 0.08) nor feeding status (fed vs. unfed) (*χ* ^2^ = 0.10, DF = 1, *p* = 0.751) significantly affected male recovery time following exposure to 0°C. However, repeated cold stress had a significant impact (*χ* ^2^ = 24.30, DF = 1, *p* < 0.001) on recovery time after the initial exposure (Figure 4a). Recovery time increased progressively over consecutive days, indicating that bees took longer to recover with each subsequent cold exposure. On average, across both exposure durations, recovery time increased by 3.5 min over four consecutive daily exposures.

In contrast, the survival analysis revealed a significant effect of feeding condition (*χ* ^2^ = 22.34, DF = 2, *p <* 0.001). Unfed males had a significantly higher risk of mortality than fed males (*β* = 1.58 ± 0.39 SE, hazard ratio [HR] = 4.86, *p* < 0.001), indicating that starvation substantially reduced survival. Exposure duration (1 h vs. 2 h at 0°C) did not significantly affect survival (*β* = −0.51 ± 0.33 SE, HR = 0.60, *p* = 0.121), suggesting that prolonged cold exposure alone was not a major driver of mortality (Figure 4b).

## 4 Discussion

We present the first assessment of thermal tolerance in the native, solitary ground‐nesting, spring bee *C. inaequalis* . Our results show that males and females exhibit similar CT_Min_ and CT_Max_ estimates, as well as comparable recovery times following a 2‐h exposure to 0°C. In addition, neither ITD nor body mass significantly influenced any of these thermal traits (Figures 2 and 3). When compared to honey bees, *C. inaequalis* shows significantly greater cold tolerance but lower heat tolerance. Contrary to our expectations, male *C. inaequalis* did not exhibit greater cold tolerance than females, nor did we find a significant effect of body size on thermal tolerance. However, our results do support the hypothesis that *C. inaequalis* is more cold‐tolerant but less heat‐tolerant than honey bees.

Our findings have important ecological and evolutionary implications. The absence of significant thermal tolerance differences between males and females, despite variations in body size and emergence timing, suggests that both sexes may experience similar selection pressures. This could indicate that both males and females must operate under the same temperature extremes throughout their brief adult lifespan. At our study site, low ambient temperatures (below ~5°C) are common even in late April, when both sexes are active (Figure S1d). In addition, *C. inaequalis* likely overwinter as adults within brood cells in the ground (Batra 1980), where both sexes may be exposed to similar thermal conditions. This shared developmental environment could further contribute to the observed similarities in thermal tolerance between males and females. The greater cold tolerance but lower heat tolerance of *C. inaequalis* compared to honey bees suggests a physiological adaptation to early spring activity and to the cool and relatively constant soil temperatures at the typical depth of brood cells of this species (Batra 1980; D.R.S., personal observations). Our measurements of the operative temperature support this idea, as these show that the thermal environment experienced by *C. inaequalis* frequently approaches or falls below CT_Min_ while the maximum operative temperatures rarely approach or exceed CT_Max_ (Figure S2). The frequent occurrence of low temperatures near or below CT_Min_ suggests that thermal constraints may be a regular challenge for *C. inaequalis* during the springtime. This could explain the low CT_Min_ values recorded for *C. inaequalis* , which are comparable or even lower than those documented for other large‐bodied, cold‐adapted taxa, such as bumble bees (e.g., Oyen et al. 2016; Oyen and Dillon 2018; Gonzalez, Oyen, Aguilar, et al. 2022).

It is important to note that *C. inaequalis* has a much broader thermal breadth (difference between CT_Max_ and CT_Min_) than honey bees (37.7–39.7 vs. 35.4°C), suggesting that this solitary species can operate across a wider range of temperatures, potentially enhancing foraging and reproductive opportunities during its narrow seasonal window. This thermal capacity is consistent with the early spring conditions, which fluctuate greatly within and among days (Figure S1d). In contrast, the narrower thermal breadth of honey bees indicates their reliance on behavioral and social thermoregulatory mechanisms to cope with temperature extremes, instead of individual physiological traits. For example, honey bees can increase nest temperature via metabolic heat production and reduce the number or duration of foraging trips to avoid extreme temperatures (e.g., Naumchik and Youngsteadt 2023; Ostwald et al. 2024). However, despite this wide thermal breadth, the lower heat tolerance relative to honey bees indicates that *C. inaequalis* may be more susceptible to rising temperatures and extreme heat events, particularly during mid‐ to late spring, potentially constraining their activity windows, reducing foraging success, and threatening their survival in a warming climate. Future studies should examine whether these bees adjust their foraging time as temperatures increase throughout the spring, as observed in some species of *Andrena* (Michener and Rettenmeyer 1956).

Thermal tolerance tends to increase with body size both within and among species in several insect groups (e.g., Baudier et al. 2018; Cerdá and Retana, 1997; González‐Tokman et al. 2021; Janowiecki et al. 2020; Oyen et al. 2016). However, this relationship appears to be more complex among bees, as it varies within species, clades, and communities. While some studies support a positive relationship between thermal tolerance and body size (Oyen et al. 2016, 2021; Gonzalez et al. 2024; Boustani et al. 2024), others find no effect of body size on either cold or heat tolerance (e.g., Oyen and Dillon 2018; Gonzalez et al. 2023). Positive correlations between body size and thermal tolerance have been observed in solitary bees of the genera *Centris* (Barrett et al. 2023) and *Eucera* (Jones et al. 2024), but not in small carpenter bees (*Ceratina*: DeHaan et al. 2025) or large carpenter bees (*Xylocopa*: Gonzalez et al. 2020). In addition, a negative correlation between body size and heat tolerance has been documented in *Osmia* (Gudowska and Morón 2024), as reported in other insects (Bota‐Sierra et al. 2022). Therefore, the lack of a significant relationship between body size and thermal traits in *C. inaequalis* falls within the range of responses observed in bees. This suggests that variations in thermal limits relative to body size may be species‐specific and that other factors, such as hydration status, cuticular modifications, or lipid content, could play a more influential role in determining thermal tolerance. In addition, these results suggest that smaller and larger individuals are equally capable of withstanding variable temperatures during the springtime, and that foraging or mating opportunities outside the nests are likely influenced by other factors, such as population density, competition, or floral availability, rather than body size‐related thermal constraints.

Our assays exploring whether lack of food and repeated or prolonged cold exposure would impair *C. inaequalis* males' ability to recover from chill coma revealed that only repeated cold exposure significantly impacted recovery time (Figure 4a), while feeding status was the primary factor influencing mortality (Figure 4b). These findings also partially agree with our initial expectations, as not all stressors had a significant impact on bees' recovery time or survival. The observed increase in recovery time, by an average of 3.5 min over four consecutive daily exposures, suggests that repetitive cold exposure increases vulnerability to low temperatures, likely due to cumulative cellular damage or metabolic depletion (e.g., Ransberry et al. 2011; Marshall and Sinclair 2012; Bayley et al. 2018). While males can tolerate short‐term cold exposure, their ability to recover deteriorates with repeated exposures. In contrast, starvation, rather than cold exposure duration, was the primary factor driving male mortality. Indeed, unfed males had a 4.86 times higher risk of mortality than fed males, as indicated by the hazard ratio value in our survival analysis. These results highlight the importance of energy reserves for tolerating cold stress and the critical role of food availability for survival in seasonal thermal environments.

Climate change is expected to increase the frequency, duration, and intensity of extreme events, such as cold snaps in early spring, and contribute to phenological decoupling between plants and pollinators (e.g., Forrest 2015; Gillespie et al. 2016; Soroye et al. 2020). Our results suggest that repeated cold exposure might impair the ability of *C. inaequalis* to resume normal activity, such as searching for mates or food, potentially affecting reproductive success. If floral resources are unavailable, starvation may further exacerbate mortality. Although we did not examine the effects of these stressors on females, they are likely to be similar given their comparable thermal tolerance.

Considering that *C. inaequalis* is physiologically adapted to early spring activity, it is likely that other solitary spring bees, including *C. thoracicus* , *C. validus* (Batra 1980), as well as certain *Andrena* and *Osmia* species, exhibit similar tolerance to low temperatures. Behavioral data support cold tolerance in some *Andrena* and suggest comparable thermal limits between sexes (Michener and Rettenmeyer 1956), as documented here for *C. inaequalis* . In addition, both field and laboratory experiments provide further evidence of greater cold tolerance in *Andrena* (Herrera et al. 2023). Unlike *C. inaequalis* , honey bees, which forage from early spring through late fall, lack comparable cold tolerance, likely relying instead on behavioral thermoregulation and social strategies (e.g., fanning) to cope with extreme temperatures. Our findings contribute to our understanding of how solitary and social bees differ in their thermal limits, providing insights into how bee distributions and community composition may shift in response to climate change. Given that most bees are solitary, we predict that bee communities, on average, will exhibit seasonal thermal tolerances that align with their activity periods, with species active in early spring displaying greater cold tolerance, while those flying in summer and fall exhibiting adaptations to warmer conditions.

## Author Contributions

**Victor H. Gonzalez:** conceptualization (equal), data curation (equal), formal analysis (equal), funding acquisition (equal), investigation (equal), methodology (equal), project administration (equal), validation (equal), visualization (equal), writing – original draft (equal), writing – review and editing (equal). **Natalie Herbison:** data curation (equal), investigation (equal), writing – review and editing (supporting). **Andres Herrera:** investigation (equal), methodology (equal), resources (equal), writing – review and editing (equal). **Kennan Oyen:** conceptualization (equal), data curation (equal), funding acquisition (equal), methodology (equal), project administration (supporting), resources (equal), software (equal), supervision (equal), validation (equal), visualization (equal), writing – review and editing (equal). **Deborah R. Smith:** conceptualization (equal), data curation (equal), funding acquisition (equal), investigation (equal), methodology (equal), project administration (equal), resources (equal), supervision (equal), validation (equal), writing – review and editing (equal).

## Conflicts of Interest

The authors declare no conflicts of interest.

## Supporting information


**Data S1:** ece371983‐sup‐0001‐Supinfo.pdf.

## Data Availability

The complete datasets used for the analyses in this study are available on Dryad: https://doi.org/10.5061/dryad.n02v6wx82.
