# Supplementary material for: Thermal Tolerance in the Cellophane Bee Colletes inaequalis Reflects Early Spring Adaptation and Is Independent of Body Size and Sex
Source: Ecol Evol. 2025 Aug 12;15(8):e71983. doi: 10.1002/ece3.71983 (PMC12340605; doi:10.1002/ece3.71983)

Supplementary Material

Thermal tolerance in the cellophane bee *Colletes inaequalis* reflects early spring adaptation and is independent of body size and sex

Victor H. Gonzalez[1*], Natalie Herbison[1], Andres Herrera[1], Kennan Oyen[2], and Deborah R. Smith[1]

[1] Department of Ecology and Evolutionary Biology, University of Kansas, Lawrence, Kansas, 66045, U.S.A.

[2]Animal Diseases Research Unit, Agricultural Research Service, United States Department of Agriculture, Pullman, Washington, 99164, U.S.A.

*Author for correspondence (vhgonza@ku.edu)

Victor H. Gonzalez: https://orcid.org/0000-0002-4146-1634

Natalie Herbison: https://orcid.org/0009-0009-8961-4503

Andres Herrera: https://orcid.org/0000-0002-7335-7049

Kennan Oyen: https://orcid.org/0000-0002-5170-6500

Deborah Smith: https://orcid.org/0000-0002-2581-5009

Running title: Thermal tolerance of an early spring bee

Figure S1. Ambient temperature and relative humidity at two nesting sites of *Colletes inaequalis* on the University of Kansas campus. Temperature and humidity were recorded every 30 minutes over 41 consecutive days, from March 30 to May 9, 2023. **a, b.** Ambient temperature and relative humidity, with trend lines representing mean values and shaded areas indicating standard deviations. **c.** Relationship between ambient temperature and relative humidity, with the trend line representing linear regression and shaded areas indicating 95% confidence intervals. **d.** Daily mean temperature, highlighting days with temperatures below 5 °C (blue) and above 25 °C (red) during the monitoring period.

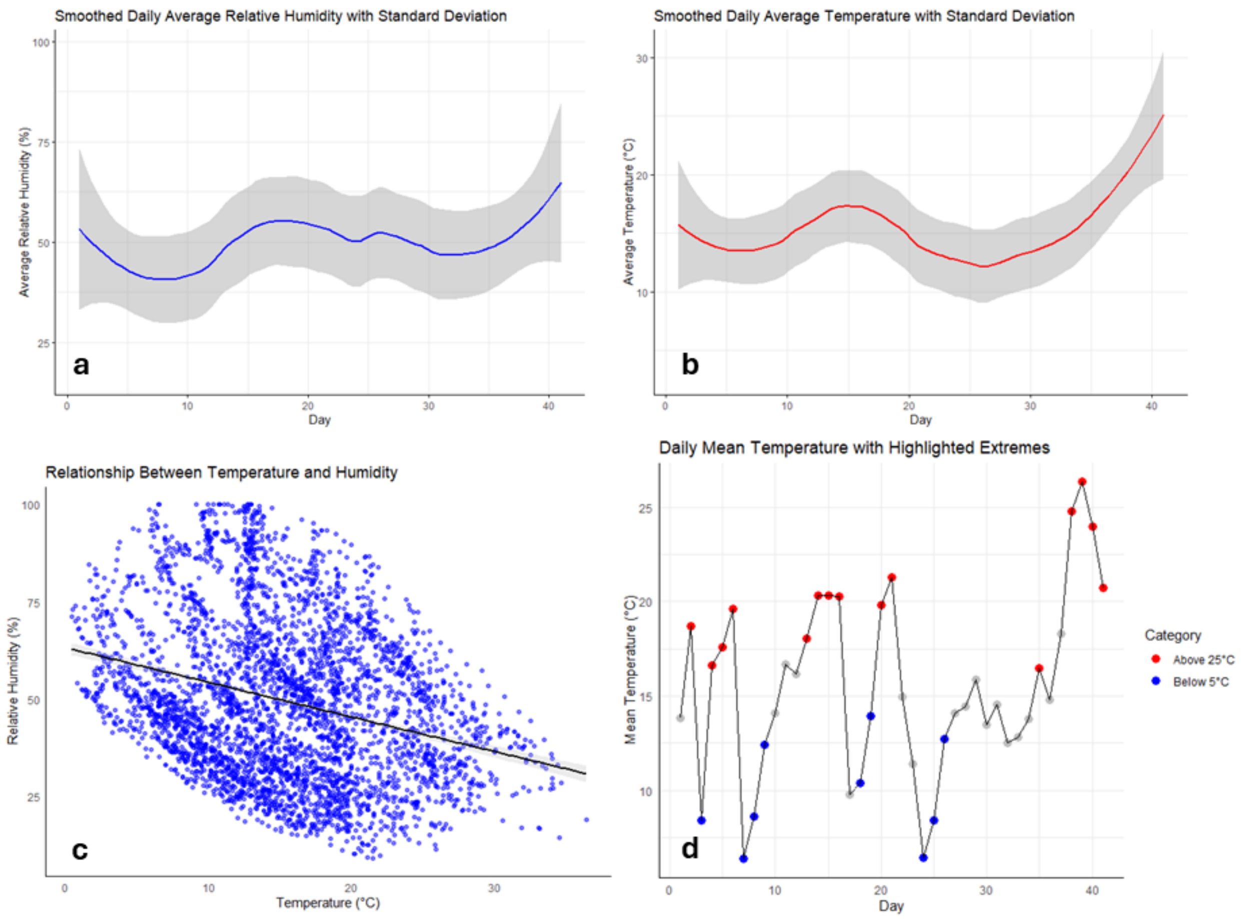

Figure S2. Operative and ambient temperatures recorded every second over five consecutive days starting on March 15 of 2024. We used a dead, dried male of *C. inaequalis* as operative temperature model. Horizontal dashed lines indicate average estimated value of the critical thermal minimum (CT_{Min} = 4.69 °C) and maximum (CT_{Max} = 44.36 °C).

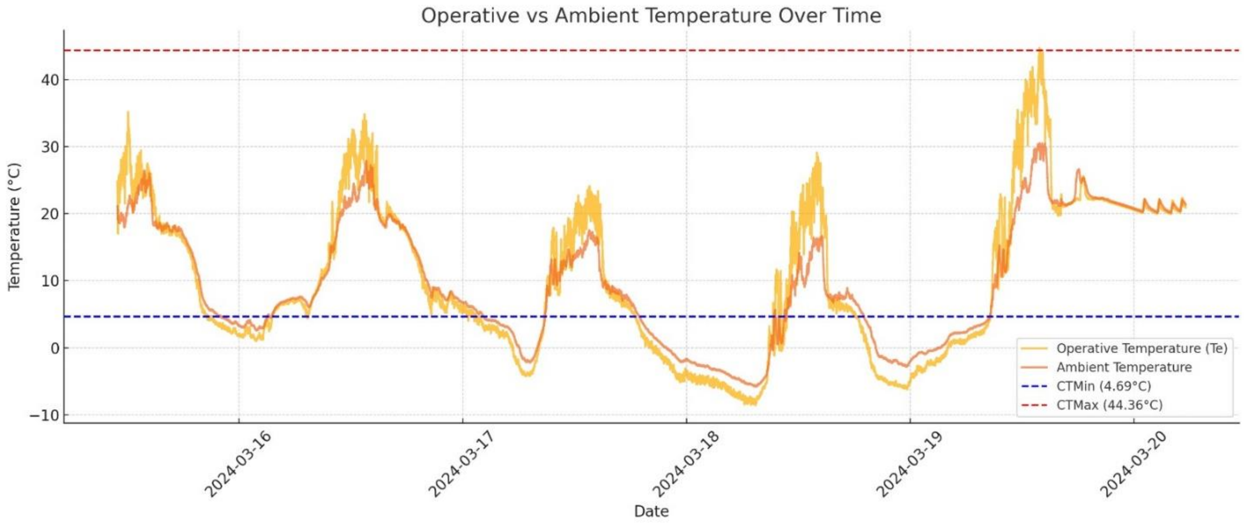

Supplement: Supplementary file 1 — Data S1: ece371983‐sup‐0001‐Supinfo.pdf. [file ECE3-15-e71983-s001.pdf]
